# Supplementary material for: The relationship between response dynamics and the formation of confidence varies across the lifespan
Source: Front Aging Neurosci. 2022 Dec 15;14:969074. doi: 10.3389/fnagi.2022.969074 (PMC9799236; doi:10.3389/fnagi.2022.969074)
Supplement: Supplementary file 1 [file Data_Sheet_1.docx]

Supplementary Material

# Sample characteristics

| **Supplementary Table 1.** | | | | | | | | |
| --- | --- | --- | --- | --- | --- | --- | --- | --- |
| *Scores of neuropsychological tests for the final sample (N = 65) and correlations with age.* | | | | | | | | |
|  | | *M* | | *SEM* | | *r* | | *p* |
| Edinburgh Handedness Inventory (EDI) | | 89.4 | | 1.3 | | -0.01 | | .938 |
| Beck’s Depression Inventory (BDI) | | 4.3 | | 0.5 | | 0.11 | | .366 |
| Mini-Mental Status Examination (MMSE) | | 29.0 | | 0.1 | | 0.04 | | .773 |
| *Note: M, mean; SEM, standard error of the mean* |  | |  | |  | |  | |

# Overview of task performance

| **Supplementary Table 2.** | | | | | | |
| --- | --- | --- | --- | --- | --- | --- |
| *Regression coefficients (β) and p-values for the predictor age for the variables mean confidence, response time (RT), and peak force (PF), and standard deviation (SD) for RT and PF.* | | | | | | |
|  | |  | | | *β* | *p* |
| Error rate [%] | | 15.4 | | | 0.005 | **< .001** |
|  | Error | *β* | *p* | Correct | *β* | *p* |
| Confidence | 2.3 | 0.019 | **< .001** | 3.8 | -0.007 | **< .001** |
| Mean RT [ms] | 734.3 | 2.568 | **.002** | 709.2 | 3.115 | **< .001** |
| SD RT [ms] | 13.9 | 0.053 | .837 | 11.5 | 0.477 | **.008** |
| Mean PF [cN] | 191.7 | 0.734 | 0.254 | 236.2 | 0.120 | .884 |
| SD PF [cN] | 10.3 | 0.137 | .774 | 13.1 | 0.164 | .704 |

# Figures control analyses

In order to get an overview of the distribution of the behavioral parameters of interest independent of confidence, we compared confidence ratings, RT, and PF between errors and correct responses. We assessed the effect of age on the response parameters RT and PF. Results of *t*-tests and simple linear regressions are reported in the manuscript. Here, we are additionally providing the respective plots for illustration purposes.


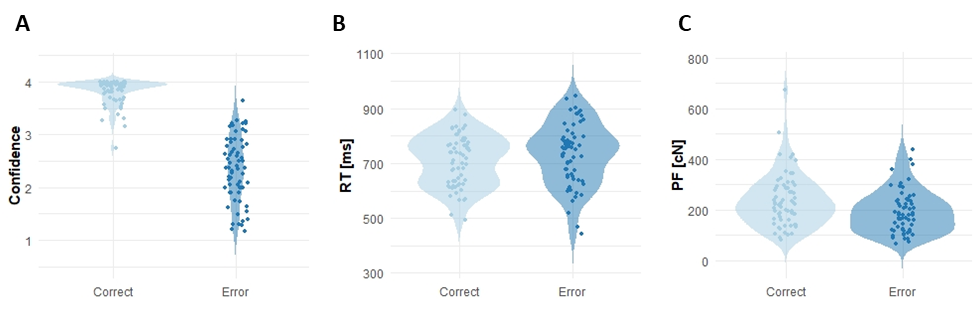


**Supplementary Figure 1.** Distribution of (A) confidence ratings, (B) RT, and (C) PF for errors and correct responses. Dots indicate individual means (for confidence) or medians (for RT and PF), respectively.


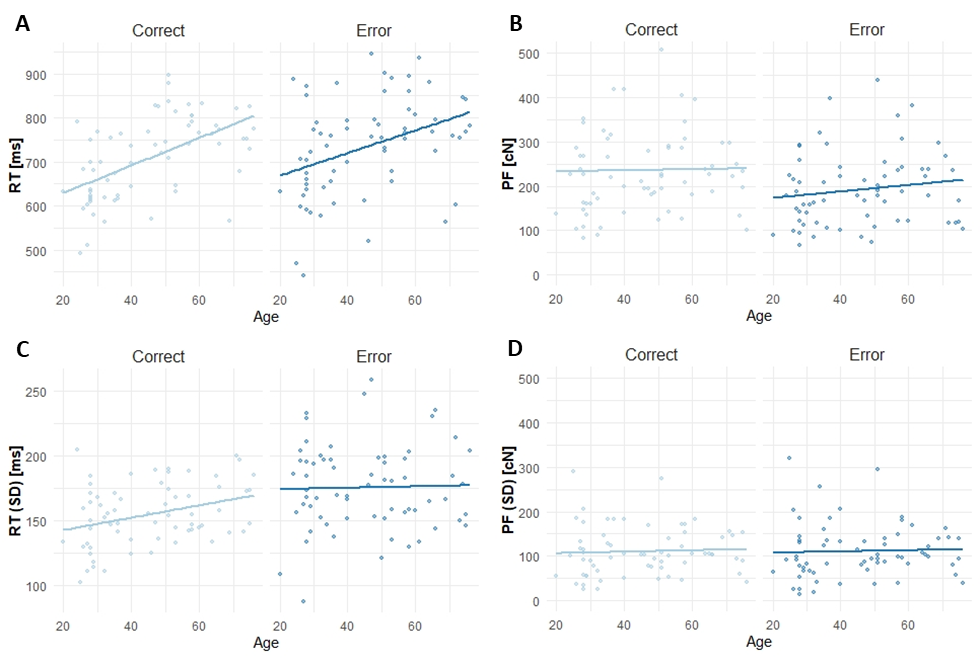


**Supplementary Figure 2.** Regression of RT (left column) and PF (right column) on age for correct and error trials, respectively. Dots and fitted lines in the upper row indicate individual median RT/PF, and dots and fitted lines in the lower row indicate individual standard deviation (*SD*) for RT/PF. Median RT of correct and error trials and *SD* RT of correct trials increase significantly with age.
